# Effects of exercise training on Fetuin-a in obese, type 2 diabetes and cardiovascular disease in adults and elderly: a systematic review and Meta-analysis

**DOI:** 10.1186/s12944-019-0962-2

**Published:** 2019-01-22

**Authors:** Robinson Ramírez-Vélez, Antonio García-Hermoso, Anthony C. Hackney, Mikel Izquierdo

**Affiliations:** 1 Centro de Estudios para la Medición de la Actividad Física CEMA, Escuela de Medicina y Ciencias de la Salud, Universidad del Rosario, Bogotá, 111221 Colombia; 2 Laboratorio de Ciencias de la Actividad Física, el Deporte y la Salud, Facultad de Ciencias Médicas, Universidad de Santiago de Chile, USACH, 7500618 Santiago, Chile; 3 Department of Exercise and Sport Science, University of North Carolina, Chapel Hill, North Carolina USA; 4 Department of Health Sciences, Public University of Navarre, CIBERFES (CB16/10/00315), Pamplona, Navarre Spain

**Keywords:** Fetuin-a, Obesity, Type 2 diabetes, Metabolic syndrome, Cardiovascular disease, Exercise training, Meta-analysis, Systematic review

## Abstract

**Background:**

Elevated levels of fetuin-A are associated with increased risks of metabolic syndrome, type 2 diabetes and nonalcoholic fatty liver disease. This meta-analysis investigated whether exercise interventions can reduce fetuin-A in adults.

**Methods:**

We searched clinical trials that objectively assessed fetuin-A and included study arms with exercise intervention. The pre-intervention and post-intervention data were used for meta-analysis. The effect sizes were calculated as standardized mean differences or changes in fetuin-A and expressed as Hedges’ g using random-effects models.

**Results:**

The overall Hedges’ g for fetuin-A in all included interventions was − 0.640 (95%CI − 1.129 to − 0.151; *n* = 9), but this effect was not observed in obese (g = − 0.096; 95%CI, − 0.328 to 0.135) and type 2 diabetes/dysglycemia (g = − 0.56; 95%CI, − 1.348 to 0.236) individuals. Additionally, the random-effects meta-regression analysis showed that there was not a greater decrease in fetuin-A in individuals who achieved greater body mass index reductions (regression coefficient = 0.065; 95%CI, − 0.185 to 0.315).

**Conclusion:**

Supervised exercise is associated with reductions in fetuin-A levels in adults and elderly. However, the results of the present meta-analysis should be interpreted with caution because of the variety of type of exercises and individual obesity related-disorders involve. Therefore, additional high-quality randomized controlled trials describing the effect of supervised exercise interventions on fetuin-A in adults are still needed.

**Electronic supplementary material:**

The online version of this article (10.1186/s12944-019-0962-2) contains supplementary material, which is available to authorized users.

## Background

Human fetuin-A (formerly named α2-Heremans-Schmid glycoprotein) is a 64-kDa glycoprotein that is found in relatively high concentrations in serum (300–1000 μg/ml) [1]. Fetuin-A is mainly expressed and secreted from the liver and adipose tissue [2]. Recent studies suggest that the liver may control whole-body energy homeostasis through the regulation of glucose and lipid metabolism by the secretion of fetuin-A [3, 4]. For example, in an animal model of diet-induced obesity that is commonly associated with hepatic steatosis, an increase in fetuin-A mRNA expression was observed in the liver [4]. Cross-sectional [5, 6] and large cohort studies [7, 8] have demonstrated consistently that elevated fetuin-A levels are associated with increased risks of subclinical and clinical cardiovascular disease (CVD).

Circulating levels of fetuin-A are increased in obesity and related disorders such as the metabolic syndrome, type 2 diabetes, and myocardial infarction/stroke [9–12]. Fetuin A stimulates the production of pro-inflammatory cytokines from adipocytes and macrophages [13] and acts as an endogenous ligand for Toll-like receptor 4, which enables free fatty acids to activate Toll-like receptor 4 signaling to induce insulin resistance [14]. Moreover, fetuin-A levels is also associated with type 2 diabetes risk [9] due to been shown to inhibit skeletal muscle insulin receptor tyrosine phosphorylation and reduce Akt activity, which in turn, contributes to decreased peripheral glucose uptake [15]. Interestingly, this liver-derived protein is also associated with fatty liver and correlates with non-alcoholic fatty liver disease (NAFLD) in humans [16].

Recent research studies have investigated the mechanisms that underlie the relationship between fetuin-A and subclinical-clinical CVD-related complications [17, 18]. Trepanowski et al. reported fetuin-A is involved in the mechanism regulating the insulin downstream signaling pathway and acts as an inhibitor of insulin resistance in muscle, liver and fat [11]. Fetuin-knockout mice exhibit improved glucose and insulin tolerance and are resistant to high-fat diet-induced weight gain [19]. Although the regulation of fetuin-A synthesis is not completely understood, its strong association within metabolic diseases has made it an attractive target for the development of novel research approaches related to metabolic health, such as insulin sensitivity, glucose tolerance or circulating lipid level treatments.

In particular, fetuin-A is the contributor which plays a critical role in the impairment of two metabolic sensors, Sirtuin 1 and AMP-activated protein kinase, in inflamed adipocytes of high fat diet mice [20]. Several studies was proposed that increased circulating fetuin-A in humans after a chronic exercise program may promote increased weight loss and improved metabolic control through elevation of adiponectin expression [20] and decrease of inflammatory cytokines in the liver and muscle [21] via fetuin-A inhibition through the AMP-activated protein kinase-nuclear factor kappa-light-chain-enhancer of activated B cells pathway [4]. Furthermore, fetuin-A directly correlated with two cardiometabolic risk markers, Apo B and C-reactive protein that, together with insulin resistance, are important components of the metabolic syndrome. This hypothesis seemed plausible, as studies demonstrated fetuin-A lowered expression of stimulated oxygen consumption and had an inverse association with blood postprandial insulin, c-peptide, and lipid peroxidation levels [21] and a positive association with adiponectin concentrations [22].

The exact role of exercise in regulating circulating fetuin-A concentration remains to be fully established. As a possible biological link between physical exercise and fetuin-A levels, cardiorespiratory fitness and muscular strength are inversely associated with liver fat and abdominal obesity [23, 24], and fetuin-A is associated with liver fat accumulation in humans [25]. A few studies have assessed the effects of lifestyle interventions such as hypocaloric diets [26] physical exercise [27, 28] or combined interventions [29] on fetuin-A, showing contradictory results. Some biological alterations promoting the protective effects of exercise on insulin sensitivity may be explained by changes in circulating fetuin-A and free fatty acids, supporting less toll-like receptor 4 signaling in adipose tissue perhaps by modulating adipose tissue macrophages [30]. In general, results on the effects of exercise on circulating fetuin-A have been rather ambiguous; diverse exercise amount and intensity within interventions may explain the highly discrepant results thus far.

In 2015, Trepanowski et al. [31] published the first comprehensive review to exclusively focus on the relationship between fetuin-A and obesity. To the best of the investigative team’s knowledge, no systematic review, with or without meta-analysis, has analyzed the effects of exercise on fetuin-A levels in humans. Hence, the present work was undertaken [31]. Due to heterogeneity between studies in terms of results, we used a meta-analytic approach to determine the effectiveness of supervised exercise interventions on fetuin-A in adults.

## Methods

### Protocol and registration

The study was undertaken in accordance with the Preferred Reporting Items for Systematic Reviews and Meta-Analyses (PRISMA) statement [32]. The review was registered with PROSPERO (CRD42017073872) at the University of York, UK. However, no study protocol was published before the initiation of the meta-analysis. All analyses were based on aggregate data from previously published studies, and thus, no ethical approval was required.

### Eligibility criteria

The a priori inclusion criteria for this study were as follows: (1) adults aged 18 years old; (2) interventions of physical exercise without hypocaloric diet intervention; and (3) assessment of serum fetuin-A.

### Information sources

A search of the literature was performed using the electronic databases Cochrane Central Register of Controlled Trials (CENTRAL), EMBASE, and MEDLINE (all: from 1998 until 15 November 2017). The terms used were as follows: [‘Fetuin-A and ‘alpha2hsglycoprotein’ OR], [‘Exercise’ and ‘training’ and ‘physical activity’ and ‘sport’ OR].

### Search

Two researchers (AG-H and RR-V) independently carried out the search. As an example, the search strategy in MEDLINE database was as follows: “Fetuin-A and alpha2hsglycoprotein” AND “metabolic risk” OR “cardiometabolic risk” OR “type 2 diabetes” “metabolic syndrome” OR “obesity”) AND (“exercise” OR “physical activity” OR “exercise intervention” OR “training”) AND (“intervention” OR “program” OR “trial” OR “treatment” OR “pre-post study”). Additionally, the reference lists of retrieved studies were examined to identify other articles. Studies reported in languages other than English were not explored.

### Study selection

Two authors (AG-H and RR-V) independently screened the titles and abstracts of potentially eligible studies identified by the search strategy. If necessary, a third researcher (MI) was consulted. The bibliographic management software EndNote version X7.0 for Windows was used for all searches. Any differences between the two authors were discussed and, if necessary, the third author was referred to for arbitration. Reasons for exclusion of identified articles were recorded in all cases.

### Data collection process and data items

Two researchers (AG-H and RR-V) independently abstracted all data. For each study, data were extracted regarding the first author’s last name, year of publication, characteristics of the subjects, exercise programs (type, frequency, duration, intensity, etc.), assessment of fetuin-A, sample size, and mean values with corresponding standard deviations of fetuin-A and BMI (pre- and post-test). The reviewers created a study-specific database in Excel (Microsoft Corp., USA) for data collection. Any differences between the two authors were discussed and, if necessary, the third author was referred to for arbitration.

### Risk of bias of individual studies

The methodological quality of non-RCT studies and studies without a control group was assessed using the Quality Assessment Tool for Quantitative Studies of the Effective Public Health Practice Project (EPHPP) [33]. This tool is used to evaluate a variety of intervention study designs, such as non-RCT or pre-post studies [33]. EPHPP assesses study quality in six domains: selection bias, study design, confounders, blinding, data collection method, and withdrawals/dropouts. Each study was assessed for quality by 2 reviewers (AG-H and RR-V), and any discrepancy in the final grade was resolved through group discussion or discussion among the primary authors.

### Summary measures

All analyses were carried out using Comprehensive Meta-analysis Software (second version, Biostat, Englewood, NJ, USA) to calculate the standardized mean difference and expressed as Hedges’ g to correct for possible small-sample bias [34]. For studies that were randomized and non-randomized controlled trials, the Hedges’ g was calculated taking the change outcome difference between the exercise and control groups and then dividing that difference by the pooled standard deviation of the change outcome difference. For single group studies, the Hedges’ g value of the fetuin-A was calculated by the mean pre- to post-intervention in each study (i.e., in all cases the pre-intervention and the post-intervention data were used to meta-analyze), dividing the result by the pooled standard deviation, and correcting for small sample bias. We used the continuous random-effect analysis with the DerSimonian-Laird method to pool results. For studies where means and standard deviations were not reported and could not be obtained from the authors, we transformed dichotomous data into the standardized mean difference using the formulas implemented in Comprehensive Meta-analysis [35] or used other statistics, such as t-values or exact *p*-values to calculate the standardized mean difference. In the cumulative meta-analysis, outcome data for fetuin-A from all available studies were included sequentially according to the year in which they first became available.

### Synthesis of results

Heterogeneity between trial results was tested with Cochran’s Q test [34] and the I^2^ statistic. I^2^ values of < 25%, 25–50, and > 50% are considered to represent small, medium, and large amounts of inconsistency [36]. Each study was deleted from the model once to analyze its influence on the overall results.

### Risk of bias across studies

Small-study effects was assessed in two ways: 1) visual inspection of the asymmetry of the funnel plot and Egger’s test of the intercept to test the symmetry of the funnel plot [37].

### Additional analysis

Subgroup analyses were conducted to determine whether fetuin-A differed according to population characteristics by stratifying the meta-analyses by each of these factors (i.e., healthy, type 2 diabetes/dysglycemia and obese) using the random-effect model. Additionally, random-effects meta-regression analyses were used to evaluate whether the results differed with BMI changes (as Hedges’ g) [38]. The effect of individual studies on the pooled Hedges’ g was assessed with influence analysis, in which the analysis was repeated omitting one study at a time, to establish the contribution of each study to the effect size.

### Patient involvement

Due to the nature of the study, no participants were involved in the systematic review and meta-analysis. Furthermore, no patients were involved in the development of the research question or outcome measures, nor were they involved in the design, implementation, recruitment, or conduct of the study. Finally, no patients were asked to advise on the interpretation or writing up of the results. There are no plans to disseminate the results of the research to the study participants.

## Results

### Study selection

**Fig. 1:**
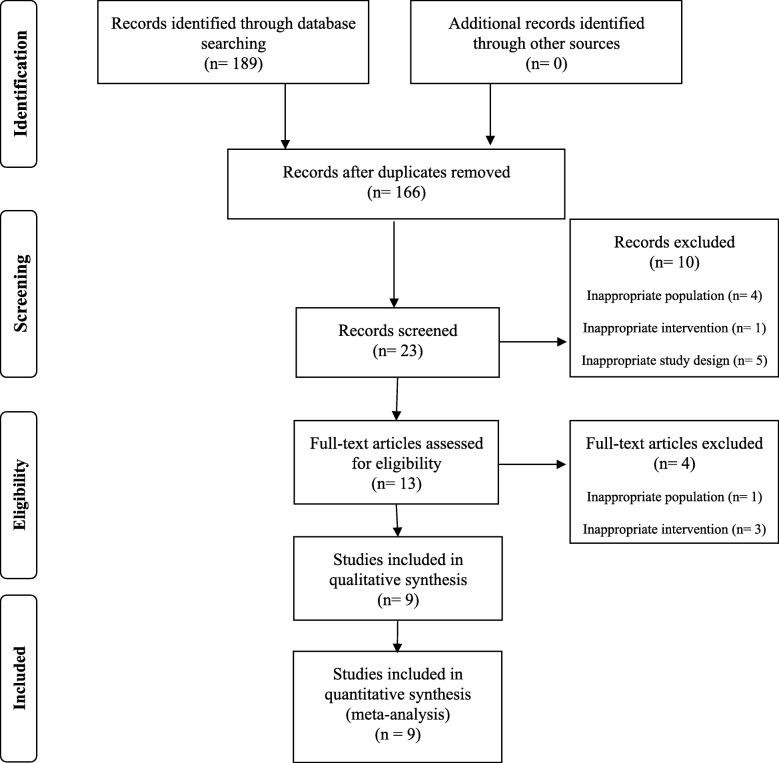
Flow chart for the identification of the meta-analyses included in the study

A total of 189 studies were identified through the database search. The titles and abstracts of the returned articles were examined for suitability, leading to the retrieval of 23 full texts. Of those 13, 4 were rejected—3 because of the type of intervention criterion (interventions with hypocaloric diet), and one because of the population issues (adolescents). Nine trials met the inclusion criteria and were included in the meta-analysis [5, 21, 27–30, 39–41]. A flow diagram summarizing the study selection process of the systematic review and meta-analysis is shown in Fig. 1. Exclusion criteria and the list of excluded articles are in online Additional file 1.

The characteristics of the nine studies included in the systematic review sorted by study design are available in Table 1. All studies were single-group pre-post design except two randomized controlled trials [40, 41] and one a randomized trial without a control group [21]. A total of 163 individuals were included in the meta-analysis. Two studies included only women [29, 39], one included only men [30], and five included mixed samples of men and women [5, 21, 27, 28, 40, 41] who were relative healthy [30], were obese [21, 27–29, 39], suffered from nonalcoholic fatty liver disease [21, 28], or had type 2 diabetes/dysglycemia or were undergoing hemodialysis [5, 30, 41].

**Table 1:** Characteristics of the included trials

Study and design	Sample	Age (years)	Type of exercise	Exercise program characteristics	Reduced Fetuin-A	EPHPP
Lee et al. 2017 [30] Single-group pre-post design	26 men (13 dysglycemic and overweight) without CVD	51.1	Concurrent	Total duration: 12 weeks Frequency a week: 4 sessions Duration per session: 60-min Aerobic; Intensity: NR Resistance; Intensity: NR	Yes	2
Malin et al. 2013 [28] Single-group pre-post design	13 obese men and women with nonalcoholic fatty liver disease without CVD	50.5	Aerobic	Total duration: 1 week Frequency a week: 5 sessions Duration per session: 60-min Intensity: 85% HRmax	Yes	2
Malin et al. 2014[27] Single-group pre-post design	20 older obese men and women without CVD	66.3	Aerobic	Total duration: 12 weeks Frequency a week: 7 sessions Duration per session: 60-min Intensity: 85% HRmax	Yes	2
Mori et al. 2008[5] Single-group pre-post design	8 men and women with type 2 diabetes mellitus	62.0	Aerobic	Total duration: 12 weeks Frequency a week: 3–5 sessions Duration per session: 40-min Intensity: 103 bpm	No	2
Schultes et al. 2010[39] Single-group pre-post design	14 obese women and without CVD	43.2	Aerobic	Total duration: 6 weeks Frequency a week: 3 sessions Duration per session: 60-min Intensity: 60% VO2peak	No	3
Wilund et al. 2010[40] RCT	17 hemodialysis men and women	59.9	Aerobic	Total duration: 16 weeks Frequency a week: 3 sessions Duration per session: 45-min Intensity: rate of perceived exertion of 12–14	No	4
Winn et al. 2017 [21] Randomized trial without a control group	18 obese men and women without CVD	51.4	Aerobic	Total duration: 4 weeks Frequency a week: 4 sessions Duration per session: NR Intensity: 55% VO2peak or 80% VO2peak	No	5
Yang et al. 2011[29] Single-group pre-post design	40 obese women without CVD	45.3	Concurrent	Total duration: 12 weeks Frequency a week: 5 sessions Duration per session: 65-min Aerobic; Intensity: 60–75% HRmax Resistance; Intensity: NR	No	2
Zhang et al. 2017 [41] RCT	32 men and women with type 2 diabetes mellitus	47.2	Aerobic	Total duration: 12 weeks Frequency a week: 5 sessions Duration per session: 70-min Aerobic; Intensity: 70% HRmax	Yes	4

*Bpm* beats per minute, *HRmax* maximum heart rate.The methodological logical quality of non-randomized controlled trials and studies without control group (*N* = 8) was assessed using the Quality Assessment Tool for Quantitative Studies of the Effective Public Health Practice Project (EPHPP) (6 evaluation critical methodological components). CVD, cardiovascular disease; RCT, randomised controlled trial

Relative to exercise programs, all studies used aerobic exercise except two that used a combined aerobic plus resistance exercise protocol (concurrent exercise) [29, 30]. Overall, the programs mainly used treadmill walking/running, cycle ergometer cycling and whole-body resistance training. The study durations ranged from one to 16 weeks, and the training frequency ranged from three to seven times weekly with 40–70 min session duration. All exercise interventions were supervised.

### Measurement of fetuin-a

All studies measured fetuin-A using enzyme-linked immunosorbent assays (ELISA) according to the manufacturer’s protocols.

### Risk of bias within studies

Only three trials had random allocation between groups [21, 40, 41]. All studies provided points and estimates of variability. Blinding of the participants and therapists was not possible because of the nature of the interventions. The studies’ “bias” score ranged from two to four with a mean total score of 2.62.

### Synthesis of results

**Fig. 2:**
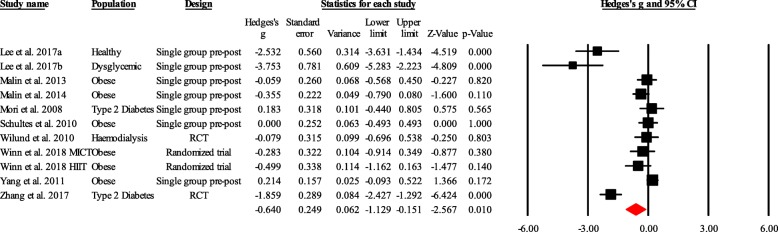
Forest plot for changes in fetuin-A. The black horizontal lines represent the 95% confidence intervals while the squares represent the Hedge’s g estimate. The first red diamond represents the overall point estimate and 95% confidence intervals from all individual studies included in each meta-analysis. All analyses are based on the random-effects model. RCT, randomized controlled trial

Overall, supervised exercise training was associated with a significant reduction in the random-effects models (g = − 0.640; 95% CI, − 1.129 to − 0.151; p = 0.010) with high heterogeneity (I^2^ = 87.60%) (Fig. 2).

### Risk of bias across studies

**Fig. 3:**
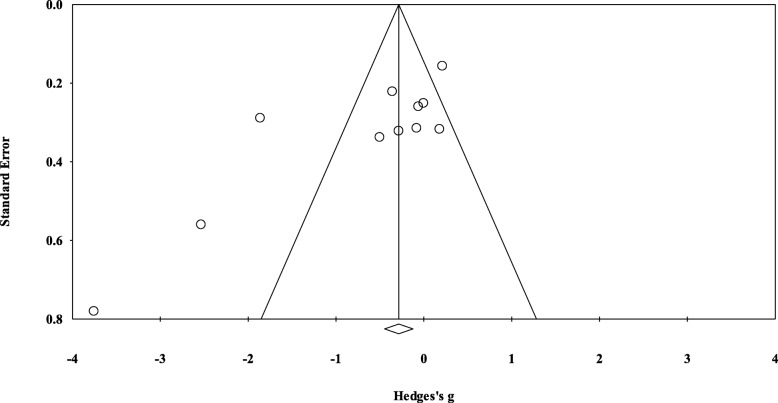
Funnel plot of precision by difference in means (Hedge’s g). Circles represent the Hedge’s g for each study and the diamond represents pooled Hedge’s g

Small-study effects by means of visual inspection of funnel plots was evaluated and the Egger’s regression asymmetry test. As shown in Fig. 3, the funnel plot was symmetrical and Egger’s linear regression tests provided no evidence for existence of small-study effects (Egger regression intercept, − 4.42 [95% CI, − 8.44 to 0.11, *p* = 0.052]).

### Additional analysis

**Fig. 4:**
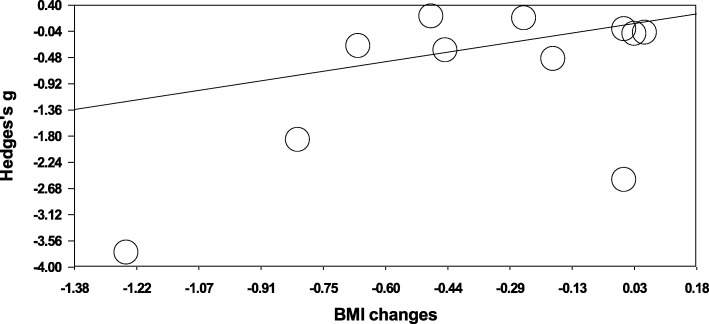
Meta-regression analysis of changes in body mass index (BMI) (X axis) against the Hedges’s g fetuin-A levels (Y axis)

Regarding subgroup analysis, in obese individuals, physical exercise favored a reduction in fetuin-A the overall same effect was not observed in individuals with obesity (g = − 0.096; 95%CI, − 0.328 to 0.135; *p* = 0.415; I^2^ = 29.04%) and type 2 diabetes/dysglycemia (g = − 1.698; 95% CI, − 3.570 to 0.174; *p* = 0.075; I^2^ = 94.24%). The independent effects of potential moderating variables were examined using meta-regression and are presented in Fig. 4. The meta-regression analysis shows that there was not a greater decrease in fetuin-A in individuals who achieved greater BMI changes. The slope regression coefficient was 0.065 [95% CI, − 0.185 to 0.315] and was not significant (*p* = 0.609).

**Fig. 5:**
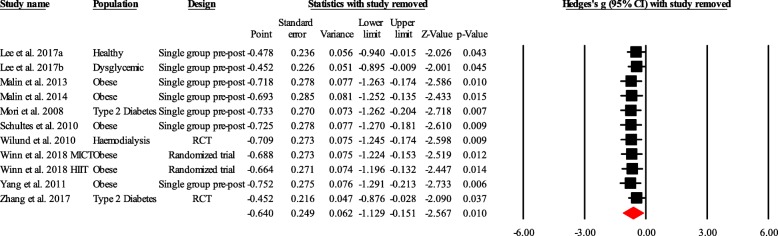
Influence analysis. RCT, randomized controlled trial

Finally, the influence analysis showed that no particular trial affected the pooled effect size (Fig. 5).

## Discussion

To our knowledge, this is the first systematic review and meta-analytic approach assessing the effects of supervised exercise training on fetuin-A in adults. The main findings of our study are as follows: (i) supervised exercise interventions is associated with reductions in fetuin-A in adults and older participants; (ii) both aerobic and resistance exercise at vigorous or moderate intensity, with a volume of 60 min/session and a minimum frequency of 4 to 7 sessions/week, significantly reduced fetuin-A levels in dysglycemic and overweight/obese individuals; and (iii) greater BMI reductions after the supervised exercise intervention are not associated with greater reductions in fetuin-A. These findings could help to provide more specific exercise recommendations for cardiometabolic risk factor management in the adult population. However, due to population heterogeneity and the high risk of bias, our findings should be interpreted with caution.

Our results show that supervised exercise training alone is associated with reductions in fetuin-A levels in adults. This finding is consistent with recently published systematic reviews and meta-analyses showing that exercise significantly modulates adipokine levels, inflammatory cytokine and glycemic control in youths [42] and patients with type 2 diabetes [30]. Jenkins et al. [43] have suggested an inverse correlation between cardiorespiratory fitness and fetuin-A, which would suggest that physical fitness level may regulate to some degree fetuin-A concentrations. However, in contrast with this conclusion, Malin et al., [27] showed that exercise-induced changes in fetuin-A were not associated with cardiorespiratory fitness in healthy adults. Two other narrative reviews [31, 44] also suggested that exercise alone might improve adipokine levels, such as fetuin-A. Our results show that supervised exercise training on fetuin-A was statistically significant when comparing post vs. pre-intervention values with large heterogeneity (I^2^ = 87.60%), even though some of the studies showed no changes after the exercise intervention. However, these results should be treated with caution given the limited number of studies included in our analysis. The differences between the results observed in the analyses examining the effect of exercise could be due to the large heterogeneity and lack of comparison with control groups. Also, these discrepancies can likely be explained by the wide range of characteristics of the participants (> 50 years old, diseases, etc.), study duration, intervention exercise program (i.e., cycling, treadmill and/or elliptical, weight machines), and extent of change in body composition across these studies.

Fetuin-A may attenuate lipogenesis and accelerate lipolysis in adipocytes, thereby promoting obesity and insulin resistance [31]. Regarding both diseases and according to subgroup analysis (individuals with type 2 diabetes/dysglycemia or obese individuals), we found no evidence to support that supervised exercise was associated with a reduction in serum fetuin-A levels. Stefan and associates conducted a study involving a complex lifestyle intervention program including dietary counseling and increased physical activity and found different results. They reported body weight decreased by ~ 3.2 kg, liver fat by 34%, and energy and, in particular, saturated fat intake by 7 and 11%, respectively, and fetuin-A levels decreased over an ~ 9-month period in subjects with a high risk for type 2 diabetes [45]. Recently, another randomized trial in obese adults confirm our findings and did not detect significant reductions in fetuin-A after applied two different exercise programs at moderate and high intensity [21]. Therefore, it is unclear whether these divergent findings are attributable to differences in the duration and intensity of the intervention, in health status, in the timing of fetuin-A measurement (e.g., within 24 h versus 36–48 h after the last exercise session), or in the sex of the participants; however, there is some evidence that relationships between fetuin-A levels and “outcomes” vs. “status” exist [45]. Reduced fetuin-A in response to intervention is often associated with improvement in insulin-related parameters [46], which was confirmed in all of the studies showing decreases in this hepatokine. For example, the study published by Lee et al. [30] demonstrated that changes in circulating fetuin-A might predict some of the benefits seen on insulin sensitivity after long-term exercise. Another study in older obese subjects also reveals that lower fetuin-A after exercise correlated with lower hepatic insulin resistance [27]. These authors suggest that fetuin-A down regulates glucose transporter-4 translocation and contributes to the improvement in skeletal muscle glucose disposal after exercise.

The meta-regression analyses showed that there was not a greater decrease in fetuin-A in individuals who achieved greater BMI changes. These findings are partly in accordance with those reported in adults with different health statuses. For example, six weeks of supervised aerobic exercise showed modest changes in body composition without affecting serum fetuin-A levels in obese older women [39]; however, 12 weeks of aerobic-exercise with significant weight loss did reduce plasma fetuin-A levels in a study of obese older men and women [28]. Additionally, a recent review suggests that weight loss seems to be effective for reducing fetuin-A level, which is not consistent with our findings [31]. Some previous studies suggest that dietary changes and weight loss [47] as well as pharmacological treatment with thiazolidinediones [5] or metformin [48] might be more potent factors than aerobic exercise alone in the regulation of hepatic fetuin-A release. Hennige et al. [49] also suggested that fetuin-A induces low-grade inflammation and represses adiponectin production in animals and in humans. Collectively, it appears likely that hepatic fat content and thus serum fetuin-A levels respond to changes in energy balance rather than to changes in body composition [39]. These data suggest an important role of fatty liver in the pathophysiology of insulin resistance and atherosclerosis. In this context, the decrease in fetuin-A observed in the present study, along with changes in BMI, could be interpreted as overall beneficial to reduce CVD risk through supervised exercise training in humans. Nonetheless, the disparate findings suggest more research and comprehensive studies of changes in fetuin-A levels as a function of exercise interventions and body composition change appears necessary.

The current meta-analytic approach was not designed to establish the exact mechanism responsible for exercise reducing fetuin-A in humans, but our data indicate that exercise is associated with lower fetuin-A, as suggested previously by others [45]. The plausible mechanisms by which supervised exercise lowers fetuin-A include the following: (i) reducing intrahepatic fat content by down-regulating sterol regulatory element-binding protein-1c and up-regulating peroxisome proliferator-activated receptor γ expression levels [50]; (ii) decreasing hepatic glucolipotoxicity through modulating the reactive oxygen species, along with inhibition of proinflammatory mediators [51]; and (iii) activating protein kinase B (also known as Akt) and Akt substrate of 160 kDa (AS160) phosphorylation, which itself has been shown to improve glucose tolerance and decrease insulin resistance [52].

The current study has several limitations. First, we included both randomized controlled trials and clinical trials, which introduced some risk of bias [53]. Second, there is a high degree of heterogeneity among the analyzed studies, in part due to the differences in inclusion criteria, health status, and the type, intensity, and duration of the supervised-exercise interventions within individual studies. Third, meta-analysis and meta-regression analyses included a reduced number of studies, some of them with small sample sizes and control groups (no intervention). A high risk of bias (i.e., quality of the studies) could be considered another limitation. Fourth, since this was an aggregate data meta-analysis, the potential for ecological fallacy exists. Fifth, since studies are not randomly assigned to covariates in meta- analysis, they are considered to be observational in nature.

Consequently, the results of subgroup and meta-regression analyses conducted in our meta-analysis do not support causal inferences and should hence be viewed as association. Large, well- designed randomized controlled trials are needed to address this issue adequately. Given the former, future randomized controlled trials may want to address some of the differences and associations observed in our current meta-analysis. In the same line, future RCT studies would need to account for baseline levels, change in other cardiometabolic traits in addition to change in BMI, and effect of medication status, alcohol and smoking patterns, and diet patterns throughout the study period. Finally, since we ran a number of analyses, some of our findings could have been nothing more than chance occurrence. Therefore, the results should be taken with caution, and more research on the effect of exercise interventions is needed to reinforce the current recommendation on exercise in the regulation of hepatic fetuin-A in humans.

## Conclusions

This meta-analysis shows that supervised exercise alone is associated with reductions in fetuin-A levels in adults and older participants. However, high quality randomized controlled trials describing the effects of supervised exercise interventions on fetuin-A in adults are few. Here in we have identify important components for future research that should be addressed as well as the limitations found in reviewing the studies for our analysis. By doing so, we hope to advance this area of research and aid in the answering of questions on exercise, hepatokines, and health, an area that is growing in interest and importance.

## Additional file

Additional file 1: List of articles excluded after full text review with reasons for exclusion. (DOCX 20 kb)

## Abbreviations

Bpm: Beats per minute
CVD: Cardiovascular disease
EPHPP: Effective Public Health Practice Project
HRmax: Maximum heart rate
PRISMA: Preferred Reporting Items for Systematic Reviews and Meta-Analyses
RCT: Randomized controlled trial
