# Supplementary material for: Effects of exercise training on Fetuin-a in obese, type 2 diabetes and cardiovascular disease in adults and elderly: a systematic review and Meta-analysis
Source: Lipids Health Dis. 2019 Jan 22;18:23. doi: 10.1186/s12944-019-0962-2 (PMC6343360; doi:10.1186/s12944-019-0962-2)
Supplement: Supplementary file 1 — List of articles excluded after full text review with reasons for exclusion. (DOCX 20 kb) [file 12944_2019_962_MOESM1_ESM.docx]

**Additional file 1**

Records excluded with reasons

1. Sargeant JA, Aithal GP, Takamura T, Misu H, Takayama H, Douglas JA, Turner MC, Stensel DJ, Nimmo MA, Webb DR, Yates T, King JA. The influence of adiposity and acute exercise on circulating hepatokines in normal weight and overweight/obese men. Appl Physiol Nutr Metab. 2017 Dec 8. doi: 10.1139/apnm-2017-0639.

Reason: Inappropriate intervention

1. Pérez-Sotelo D, Roca-Rivada A, Larrosa-García M, Castelao C, Baamonde I, Baltar J, Crujeiras AB, Seoane LM, Casanueva FF, Pardo M. Visceral and subcutaneous adipose tissue express and secrete functional alpha2hsglycoprotein (fetuin a) especially in obesity. Endocrine. 2017 Feb;55(2):435-446. doi: 10.1007/s12020-016-1132-1.

Reason: Inappropriate intervention

1. Blumenthal JB, Gitterman A, Ryan AS, Prior SJ. Effects of Exercise Training and Weight Loss on Plasma Fetuin-A Levels and Insulin Sensitivity in Overweight Older Men. J Diabetes Res. 2017;2017:1492581. doi: 10.1155/2017/1492581.

Reason: Inappropriate intervention

1. König D, Zdzieblik D, Deibert P, Berg A, Gollhofer A, Büchert M. Internal Fat and Cardiometabolic Risk Factors Following a Meal-Replacement Regimen vs. Comprehensive Lifestyle Changes in Obese Subjects. Nutrients. 2015 Dec 1;7(12):9825-33. doi: 10.3390/nu7125500.

Reason: Inappropriate intervention

1. Trepanowski JF, Mey J, Varady KA. Fetuin-A: a novel link between obesity and related complications. Int J Obes (Lond). 2015 May;39(5):734-41. doi: 10.1038/ijo.2014.203.

Reason: Inappropriate study design

1. McClellan JL, Steiner JL, Day SD, Enos RT, Davis MJ, Singh UP, Murphy EA. Exercise effects on polyp burden and immune markers in the ApcMin/+ mouse model of intestinal tumorigenesis. Int J Oncol. 2014 Aug;45(2):861-8. doi: 10.3892/ijo.2014.2457.

Reason: Inappropriate population

1. Hwang JJ, Thakkar B, Chamberland JP, Mantzoros CS. Circulating fetuin-A levels are not affected by short and long-term energy deprivation and/or by leptin administration. Metabolism. 2014 Jun;63(6):754-9. doi: 10.1016/j.metabol.2014.02.006.

Reason: Inappropriate study design

1. Sakr HF, Al-Hashem FH, El-Naby WM, Alkhateeb MA, Zaki MS, Refaey HM, Morsy MD. Preventive roles of swimming exercise and pioglitazone treatment on hepatic dysfunction in a rat model of metabolic syndrome. Can J Physiol Pharmacol. 2014 Feb;92(2):162-70. doi: 10.1139/cjpp-2013-0043.

Reason: Inappropriate population

1. Minas M, Mystridou P, Georgoulias P, Pournaras S, Kostikas K, Gourgoulianis KI. Fetuin-A is associated with disease severity and exacerbation frequency in patients with COPD. COPD. 2013 Feb;10(1):28-34. doi: 10.3109/15412555.2012.727922.

Reason: Inappropriate study design

1. Jenkins NT, McKenzie JA, Hagberg JM, Witkowski S. Plasma fetuin-A concentrations in young and older high- and low-active men. Metabolism. 2011 Feb;60(2):265-71. doi: 10.1016/j.metabol.2010.01.026.

Reason: Inappropriate study design

1. Choi, K.M., Han, K.A., Ahn, H.J., Lee, S.Y., Hwang, S.Y., Kim, B.H., et al. (2013). The effects of caloric restriction on fetuin-A and cardiovascular risk factors in rats and humans: a randomized controlled trial. Clin. Endocrinol. (Oxf). 79(3):356-363.

Reason: Inappropriate intervention

1. Matsumoto Y, Adams V, Jacob S, Mangner N, Schuler G, Linke A. Regular exercise training prevents aortic valve disease in low-density lipoprotein-receptor-deficient mice. Circulation. 2010 Feb 16;121(6):759-67. doi: 10.1161/CIRCULATIONAHA.109.892224.

Reason: Inappropriate population

1. Reinehr T, Roth CL. Fetuin-A and its relation to metabolic syndrome and fatty liver disease in obese children before and after weight loss. J Clin Endocrinol Metab. 2008 Nov;93(11):4479-85. doi: 10.1210/jc.2008-1505.

Reason: Inappropriate population

1. Zmuda JM, Eichner JE, Ferrell RE, Bauer DC, Kuller LH, Cauley JA. Genetic variation in alpha 2HS-glycoprotein is related to calcaneal broadband ultrasound attenuation in older women. Calcif Tissue Int. 1998 Jul;63(1):5-8. PubMed PMID: 9632839.

Reason: Inappropriate study design
